# Antibioprophylaxie dans les chirurgies gynécologiques et obstétricales propres et propres contaminées à l'Hôpital Général de Yaoundé, Cameroun

**DOI:** 10.11604/pamj.2014.19.23.4534

**Published:** 2014-09-10

**Authors:** Jean Dupont Kemfang Ngowa, Anny Ngassam, R Motzebo Mbouopda, Jean Marie Kasia

**Affiliations:** 1 Service de Gynécologie-obstétrique, Hôpital Général de Yaoundé, Yaoundé, Cameroun; 2 Département de Gynécologie-obstétrique, Faculté de Médecine et des Sciences Biomédicales, Université de Yaoundé I, Yaoundé, Cameroun; 3 Service d'Anesthésie, Hôpital Général de Yaoundé, Yaoundé, Cameroun

**Keywords:** Antibioprophylaxie, chirurgie gynécologique ou obstétricale, infection post opératoire, chirurgie propre, chirurgie propre contaminée, antibioprophylaxis, obstetric and gynecologic surgery, postoperative infection, clean surgery, clean contaminated surgery

## Abstract

**Introduction:**

Il s'agira de déterminer l'incidence et les facteurs associés à l'infection post opératoire dans les chirurgies gynécologiques et obstétricales propres et propres contaminées.

**Méthodes:**

Étude de cohorte prospective axée sur la surveillance de l'infection post opératoire chez les patientes opérées d'une chirurgie gynécologique ou obstétricale propre ou propre contaminée au Service de Gynécologie B de l'Hôpital Général de Yaoundé, après une antibioprophylaxie avec la cefazoline. La période d’étude de 18 mois s'est étalée de janvier 2012 à juin 2013. La surveillance des patientes opérées se poursuivait sur les 30 premiers jours post opératoires.

**Résultats:**

Au total 211 patientes opérées d'une chirurgie gynécologique ou obstétricale propre ou propre contaminée ont été enrôlées dans cette étude. La moyenne d’âge des patientes était de 35,4 ±10 ans; les chirurgies propres contaminées représentaient 76,3% et les chirurgies propres 23,7%. Nous avons observé au total 06 (2,8%) cas d'infection post opératoire, dont 03 (1,4%) cas d'infection du site opératoire, 02 (0,95%) cas d'infection urinaire et 01(0,47%) cas d'infection pulmonaire. Les variables significativement associées à l'infection post opératoire étaient la durée de la chirurgie (durée moyenne: 126± 89min vs 82± 50 min; p< 0,04) et le diabète (OR. 40,8; IC à 95%. 2,2-749,2; p < 0,000.

**Conclusion:**

Notre taux d'infection post opératoire global de 2,8% et d'infection du site opératoire de 1,4% vient renforcer les données de la littérature sur l'efficacité de l'antibioprophylaxie dans les chirurgies gynécologiques et obstétricales propres et propres contaminées en milieu africain. Cependant le respect des principes d'asepsie péri opératoire et une sélection précise de la classe de chirurgie devraient être de règle dans notre contexte.

## Introduction

Les complications infectieuses des chirurgies gynécologiques et obstétricales sont des sources potentielles de morbidité et de mortalité. Les infections post opératoires en gynécologie obstétrique comprennent l´infection des voies urinaires, l´endométrite, l´infection de la plaie opératoire, l´infection du périnée, et la septicémie, qui conduisent à des hospitalisations prolongées et une augmentation des coûts de soins de santé [1]. L'infection post opératoire représente la première cause de morbidité à la maternité du CHU de Dakar [2]. Au Cameroun, une étude menée au Centre Hospitalier et Universitaire de Yaoundé a révélé une incidence de l'infection post opératoire élevée de 23,5% dans les chirurgies gynécologiques et obstétricales [3].

L'importance de l'antibioprophylaxie dans la réduction de la morbidité infectieuse post opératoire n'est plus à démontrer [1, 4–7]. L'antibioprophylaxie s'applique aux interventions propres ou propres contaminées. Pour les interventions contaminées ou sales, l'infection est déjà en place et relève d'une antibiothérapie curative [8]. La durée de l´antibioprophylaxie doit être la plus courte possible. L'injection d'une dose unique est recommandée et la prescription au-delà de 48 heures est proscrite [8].

Cependant, la prescription d´une antibioprophylaxie est souvent inappropriée [9, 10]. En Tunisie, une étude hospitalière a révélé une fréquence élevée de 59,3% des prescriptions inappropriées d'antibioprophylaxie en péri-opératoire [9]. Dans nos pays en voie de développement, certains praticiens prescrivent une antibioprophylaxie de longue durée (7 à 10 jours) dite de « couverture » pour pallier selon eux à la rupture d'asepsie parfois constatée dans nos blocs opératoires. Cette pratique augmente considérablement le coût de la chirurgie (antibioprophylaxie prolongée = 26500 FCFA/ 40€ 45 contre 3650 FCFA / 5€ 57 pour l'antibioprophylaxie standard avec la Cefazoline) et le risque de voir se développer des résistances aux antibiotiques utilisés en curatif. Par contre, une étude hospitalière menée à Dakar au Sénégal sur l'efficacité de l'antibioprophylaxie par la cefotaxime dans les chirurgies Gynécologiques et obstétricales propres et propres contaminées a rapporté une absence d'infection post opératoire [2].

Depuis deux années, dans le service de Gynécologie B de l'Hôpital Général de Yaoundé (HGY), l'antibioprophylaxie par la Cefazoline a été instituée dans les chirurgies gynécologiques et obstétricales propres et propres contaminées. L'objectif de notre étude était de déterminer l'incidence et les facteurs associés à l'infection post opératoire dans les chirurgies gynécologiques et obstétricales propres et propres contaminées au Service de Gynécologie B de l'Hôpital Général de Yaoundé dans un contexte de pratique de l'antibioprophylaxie par la Cefazoline.

## Méthodes

Il s'agissait d'une étude de cohorte prospective portant sur une surveillance continue des infections post opératoires chez les femmes opérées d'une chirurgie gynécologique ou obstétricale propre ou propre contaminée pendant la période du 1er janvier 2012 au 30 juin 2013 dans le service de Gynécologie B de l'Hôpital Général de Yaoundé. Les anesthésistes et les gynécologues obstétriciens ont collégialement institué depuis Janvier 2011 dans le service de gynécologie B de l'HGY la pratique de l'antibioprophylaxie avec la Cefazoline dans les chirurgies gynécologiques et obstétricales propres et propres contaminées. Dans ce service, nous considérons les chirurgies propres et propres contaminées selon la classification d'Altemeier [11]. Les patientes programmées pour les chirurgies gynécologiques et obstétricales sont admises la veille de l'opération. Elles sont souvent déjà rasées et le cas contraire, elles seront rasées par l'infirmière de service avec une lame de rasoir neuve à usage unique. Le matin du jour de l'intervention, les patientes prennent une douche complète, puis elles sont transportées au bloc opératoire dévêtues et recouvertes de draps propres fournis par l'hôpital. Au bloc opératoire, le lavage chirurgical des mains se fait avec la Bétadine^®^ rouge à 4% et de l'eau potable. Le port de la casaque et des gants stériles par les opérateurs se fait dans la salle opératoire. Les champs, les casaques et les boites de chirurgie sont à usage multiples. Après chaque intervention, ils sont décontaminés, lavés et stérilisés par les unités de la Buanderie et de la Stérilisation de l’ HGY. Au début et à la fin de l'intervention, la désinfection cutanée de la zone d'incision se fait par un badigeonnage à la Bétadine^®^ dermique 10% en deux passages et la plaie opératoire est recouverte à la fin par un pansement stérile.

L'antibioprophylaxie est pratiquée avec la Cefazoline, une dose initiale de 2g est administrée par voie intraveineuse 30min avant l'incision sauf dans les césariennes où l'administration se fait après le clampage du cordon ombilical. Une réinjection d'1g de Céfazoline est administrée en per opératoire pour les chirurgies dont la durée est = 4heures. Par ailleurs, une dose supplémentaire de 1g de Céfazoline est administrée systématiquement à la 6e heure post opératoire. La surveillance post opératoire des patientes est assurée jusqu’à leur sortie de l'hôpital par les résidents de gynécologie obstétrique sous la supervision des gynécologues obstétriciens du service. A la sortie de l'hôpital, une consultation est systématiquement organisée au 30ejour post opératoire avec le gynécologue obstétricien opérateur.

Nous avons enrôlé dans l´étude toutes les patientes opérées d'une chirurgie gynécologique ou obstétricale propre ou propre contaminée pendant la période de l’étude. Nous avons considéré comme chirurgies propres les interventions sans ouverture de viscères creux, sans notion de traumatisme ou d'inflammation préalable à la chirurgie (chirurgie du sein sans drainage, chirurgie des annexes utérines sans signes d'infection; myomectomie sans ouverture de la cavité utérine). Comme chirurgies propres contaminées nous avons considéré les situations de chirurgies propres avec ouverture de viscères creux avec contamination minime ou avec une rupture minime d'asepsie (césarienne, chirurgie du sein avec drainage, hystérectomie, myomectomie avec effraction de la cavité utérine, chirurgie vaginale). Les cas de chirurgies contaminées ou sales, les césariennes après rupture des membranes de plus de 6 heures; les patientes séropositives au VIH avec un taux de CD4 inférieur à 350/ml et les patientes ayant nécessité en pré ou post opératoire un traitement avec des antibiotiques dans un cadre autre que celui d'une antibioprophylaxie étaient exclues de cette étude.

En ce qui concerne le diagnostic de l'infection post opératoire, toute suspicion par le résident était validée par le gynécologue obstétricien. Les critères de diagnostic de l'infection du site opératoire survenant dans les 30 jours suivant l'intervention étaient ceux définis par la CDC [12]. Le diagnostic de l'infection au site opératoire pariétal superficiel ou profond était basé principalement sur l’écoulement d'un liquide suppuré de la plaie ou d'un drain pariétal. Par ailleurs, le diagnostic d'endométrite post opératoire était basé sur la douleur abdomino-pelvienne associée à des lochies malodorantes ou franchement purulentes. Le diagnostic d'infection urinaire était toujours confirmé à la culture des urines. Les autres infections post opératoires telle que l'infection pulmonaire étaient basées sur les signes cliniques et radiologiques évocateurs.

Pour chaque patiente, les variables étudiées étaient: âge, poids, indice de masse corporelle (IMC), classe de la chirurgie (propre ou propre contaminée), type de chirurgie (d'urgence ou programmée), indication opératoire, les morbidités sous-jacentes (diabète, VIH, chimiothérapie neoadjuvante pour cancers, obésité morbide: IMC > 35), antibioprophylaxie, durée de l'opération, les infections post opératoires, la durée d'hospitalisation, la fièvre post opératoire, hémogramme, culture des urines, radiographie des poumons. Les données ont été saisies et analysées dans Epi Info version 3.5.1. La fréquence et le pourcentage des différentes variables ont été calculés. Nous avons calculé les Odds Ratios des variables pouvant influencer la survenue de l'infection post opératoire. Le seuil de significativité était considéré pour p < 0,05.

## Résultats

Au total, 211 patientes opérées d'une chirurgie propre ou propre contaminée au service de gynécologie B de l'HGY ont été enrôlées dans cette étude. La moyenne d’âge de ces patientes était de 35,4 ± 10 ans.

**Caractéristiques de la population étudiée:** le Tableau 1 résume les caractéristiques des patientes opérées. La grande majorité des patientes (87,7%) avait 45ans ou moins. L'obésité morbide et l'infection au VIH étaient retrouvées respectivement chez 10,9% et 4,3% des patientes opérées.

**Tableau 1:** Caractéristiques des patientes opérées

caractéristiques	Patientes opérées n = 211	
	Fréquence	%
**Tranches d’âges (années)**		
12 – 25	32	15,2
26 – 35	81	38,4
36 – 45	72	34,1
46 – 55	18	8,5
56 – 65	4	1,9
66 – 75	4	1,9
Chimiothérapie néo adjudante pour cancer	13	6,2
Diabète	2	0,9
HIV avec CD4 > 350/ml	9	4,3
Obésité (IMC >35)	23	10,9

**Répartition des patientes selon les caractéristiques de la chirurgie réalisée:** la durée moyenne des interventions chirurgicales était de 83,51± 51 minutes. Le Tableau 2 montre la répartition des patientes opérées en fonction des différentes caractéristiques de la chirurgie. Les chirurgies propres contaminées étaient plus fréquentes (76,3%) que les chirurgies propres (23,7%). Tandis que les chirurgies programmées représentaient 88,6% de toutes les chirurgies réalisées. Les césariennes et les laparotomies pour une chirurgie gynécologique étaient les plus fréquentes, respectivement 27,96% et 25,59%, suivies de la cœlioscopie opératoire (19,43%) et de la chirurgie mammaire (17,54%).

**Tableau 2:** Répartition des patientes selon les caractéristiques de la chirurgie

Caractéristiques de la chirurgie	Patientes opérées (n = 211)	
	Fréquence	%
Classe de la chirurgie		
Chirurgie propre	50	23,7
Chirurgie propre contaminée	161	76,3
Type de chirurgie		
Chirurgie d'urgence	24	11,4
Chirurgie programmée	187	88,6
Nature de la chirurgie		
Césarienne	59	27,96
Laparotomie	54	25,59
Myomectomie	30	14,22
Hystérectomie	22	10,42
Chirurgie carcinologique pelvienne	2	0,95
Cœlioscopie opératoire	41	19,43
Cœlioscopie post myomectomie	41	8,53
Cœlioscopie pour l'infertilité	18	7,11
Kystectomie ovarienne	15	2,37
Salpingectomie pour GEU	5	1,42
Cœlioscopie diagnostique	4	1,90
Chirurgie mammaire	37	17,54
mastectomie	14	6,64
Chirurgie conservatrice	18	8,53
plastie mammaire	5	2,37
Chirurgie vaginale	16	7,58
Chirurgie du col utérin*	12	5,68
Aspiration utérine	2	0,95
Plastie vulvo-vaginale	2	0,95
**Durée de la chirurgie**		
≤2heures	160	75,8
>2- 4heures	47	22,3
>4heures	4	1,9

* Cerclage utérin; conisation du col utérin

**Infections post opératoires:** nous avons observé au total 06 (2,8%) cas d'infection post opératoires, dont 03 (1,4%) cas d'infection du site opératoire, 02 (0,95%) cas d'infection urinaire et 01(0,47%) cas d'infection pulmonaire chez une patiente infectée au VIH et sous traitement par les antirétroviraux avec un taux de CD4 > 350/ml au moment de la chirurgie. Des 03cas d'infection du site opératoire, 02(0,95%) cas étaient une infection pariétale au site de l'incision cutanée et 01(0,47%) cas était une endométrite du post partum après césarienne d'urgence. Les infections post opératoires concernaient 03 cas de césarienne, 02 cas de myomectomie par laparotomie et 01 cas de mastectomie.

Nous avons enregistré 15 (7,2%) cas de fièvre (température >38°C) post opératoire, parmi lesquels 03 (20%) cas étaient associés aux infections post opératoires et 12 (80%) cas étaient des accès palustres dont l’évolution était favorable sous traitement par des antipaludéens. LeTableau 3 montre l´association de certaines variables avec l´infection post opératoire. La durée moyenne de la chirurgie était significativement plus élevée chez les patientes présentant une infection post opératoire (126± 89min vs 82± 50 min; p< 0,04). Le diabète était significativement associé à l´infection post opératoire (OR. 40,8(2,2-749,2); p < 0,000). Par ailleurs, l´âge, l´infection au VIH, la chimiothérapie néo adjuvante, l´obésité, le type de chirurgie (programmée ou urgente), la classe de chirurgie (propre ou propre contaminée) n´étaient pas significativement associés à l´infection post opératoire.

**Tableau 3:** Étude de l'association entre certaines variables et l'infection post opératoire

caractéristiques	Infection post opératoire				Odd ratio (IC à 95%)	P value
	oui		non			
	n	%	n	%		
**Age (Moyenne)**	34,50 ± 7,17 ans		35,43 ± 10,55 ans		-	0,8291
**Durée de chirurgie (Moyenne)**	126± 89 min		82± 50min		-	0,0402
**Facteurs morbides associés**						
**Diabète**						
Oui	1	50	1	50	40,8 (2,2-749,2)	0,00006
Non	5	2,4	204	97,6	1b	
**Infection VIH**						
Oui	1	11,1	8	88,9	4,9 (0,5 – 47,2)	0,1272
Non	5	2,5	197	97,5	1b	
**Chimiothérapie néo adjuvante**						
Oui	1	7,7	12	92,3	3,2 (0,4 – 29,8)	0,2776
Non	5	2,5	193	97,5	1b	
**Obésité**						
Oui	2	8,7	21	91,3	4,4(0,8 – 25,4)	0,0736
Non	4	2,1	184	97,9	1b	
**Type de chirurgie**						
Programmée	4	2,1	183	97,9	1b	
Urgente	2	8,3	22	91,6	4,2(0,7 – 24,0)	0,0857
**Classe de chirurgie**						
Propre contaminé	6	3,7	155	75,6	1b	
Propre	0	0,0	50	100	Non défini	0,1661

## Discussion

Le risque d'infection post opératoire représente un problème majeur lors de la réalisation de tout acte chirurgical. Il s'agit d'un risque permanent, en effet 90% des plaies opératoires sont contaminées des bactéries pathogènes au moment de la fermeture pariétale [10, 12]. Nous avons obtenu un taux global d'infection post opératoire de 2,8% et un taux d'infection du site opératoire de 1,4% qui se situent dans les tranches habituellement rapportées dans la littérature (< 2% pour les chirurgies propres et < 10% pour les chirurgies propres contaminées) [8, 13]. Notre étude, tout comme l’étude sénégalaise [2], confirme l'efficacité de la pratique de l'antibioprophylaxie dans les chirurgies gynécologiques et obstétricales propres et propres contaminées en milieu Africain. Cependant, notre taux global d'infection post opératoire de 2,8% est de loin inférieur au taux de 23,2% retrouvé dans les chirurgies gynécologiques au CHU de Yaoundé [3]. Le taux d'infection post opératoire plus élevé dans l’étude menée au CHU de Yaoundé peut s'expliquer d'une part par la différence dans les critères de sélection des patientes dans les deux études. L’échantillon était limité dans notre étude aux chirurgies propres et propres contaminées alors qu'il était étendu dans l’étude du CHU à tous les cas de chirurgies gynécologiques et obstétricales à l'exception des cœlioscopies, mini laparotomie pour ligature des trompes. D´autre part, cette différence pourrait aussi s'expliquer par la disparité du plateau technique dans ces deux hôpitaux. Le CHU de Yaoundé est âgé de plus de 30ans par rapport à l´HGY qui est un hôpital plus récent. En conséquence, les équipements sont vétustes au CHU et prédisposeraient à un risque plus élevé de rupture d´asepsie.

Certaines morbidités sous-jacentes chez les patientes opérées comme l'obésité, le diabète ou un état d'immunodépression sont connues comme étant des facteurs de risque d'infection post opératoire [10, 12, 14]. Le diabète et la durée de la chirurgie étaient associés significativement (respectivement p= 0,0001 et p = 0,04) à un risque plus élevé d'infection post opératoire dans notre étude. La durée moyenne de la chirurgie significativement plus longue parmi les patientes ayant eu une infection post opératoire dans cette étude, concorde bien avec les données de la littérature sur l'association du risque d'infection post opératoire avec la durée de l'intervention [10].

Contrairement aux données de la littérature [12], l'infection au VIH dans notre étude n’était pas associée à un risque d'infection post opératoire plus élevé. La raison plausible serait la sélection des patientes infectées au VIH limitée à celles ayant un taux de CD4 > 350/ml et éliminant de ce fait celles présentant un taux de CD4 < 350/ml qui sont potentiellement immuno-incompétentes. Par ailleurs, l'obésité et la chimiothérapie néo adjudante n’étaient pas significativement associées à un risque plus élevé d'infection post opératoire dans cette étude. La petite taille de l´échantillon dans ces deux facteurs de morbidité nous semble être la raison la plus vraisemblable.

L'antibioprophylaxie répond à une administration brève d'antibiotiques juste avant le début de l'opération [12, 15, 16]. Les céphalosporines sont les antibiotiques les plus indiqués dans l'antibioprophylaxie [17]. Les céphalosporines sont efficaces contre la plupart de bactéries gram négatifs et gram positifs. Ils ont un certain nombre de caractéristiques: bonne tolérance, pharmacocinétique simple, et un coût raisonnable. En particulier, la Cefazoline est largement utilisée et généralement considérée comme l'antibiotique de premier choix pour l'antibioprophylaxie dans les chirurgies propres [18]. Au service de gynécologie B de l'HGY, nous pratiquons l'antibioprophylaxie dans les chirurgies gynécologiques et obstétricales propres et propres contaminées avec la Cefazoline, ce qui concorde bien avec les données de la littérature.

La prévention des infections du site opératoire comprend l´administration d´une antibioprophylaxie appropriée, la maitrise des techniques chirurgicales, l´application rigoureuse des principes d´asepsie pour réduire les risques de contamination bactérienne, virale et fongique causée par le personnel soignant, l´environnement de la salle d´opération et la flore endogène cutanée de la patiente [12, 19]. Le respect de l'asepsie au bloc opératoire de l'Hôpital Général de Yaoundé par le personnel de chirurgie et d´anesthésie est sous la surveillance rigoureuse de l'infirmier major du bloc opératoire et du médecin chef du service de chirurgie. Par ailleurs, les opérations chirurgicales du service de gynécologie B sont effectuées par deux chirurgiens gynécologues séniors assistés par les résidents en gynécologie obstétrique, assurant ainsi une excellente pratique des techniques chirurgicales.

## Conclusion

Notre taux global d'infection post opératoire de 2,8% et d'infection du site opératoire de 1,4% viennent renforcer les données de la littérature sur l'efficacité de l'antibioprophylaxie dans les chirurgies gynécologiques propres et propres contaminées en milieu africain. Le diabète et la durée de la chirurgie sont deux facteurs significativement associés à l'infection post opératoire dans notre étude. Toutefois la sélection minutieuse des chirurgies propres et propres contaminées et le respect rigoureux des principes d'asepsie en péri-opératoire sont des facteurs pouvant influencer considérablement l'efficacité de l'antibioprophylaxie et doivent être scrupuleusement appliqués dans notre milieu. D'autre part, la pratique de l'antibioprophylaxie dans les indications appropriées réduirait considérablement le coût de la chirurgie et en conséquence favoriserait dans notre contexte l'accessibilité aux soins chirurgicaux.
